# P19 Nurse evaluation of a local antibiotic IV to oral switch (IVOS) prompt tool

**DOI:** 10.1093/jacamr/dlad066.023

**Published:** 2023-06-14

**Authors:** Sue Bowler, Annette Clarkson

**Affiliations:** Nottingham University Hospitals NHS Trust, UK; Nottingham University Hospitals NHS Trust, UK

## Abstract

**Background:**

Early IVOS has numerous benefits for our patients, our planet, and our nurses.^1,2^ Importantly, a multisite qualitative study concluded that encouraging the prompt transition from IV to oral antibiotics was perceived favourably by nurses as an antibiotic stewardship nursing role.^3^ In November 2022, the UK Health Security Agency published the national IVOS criteria;^1^ these formed the basis of our revised local IVOS prompt tool. The aim of the project was to evaluate nurses’ initial responses to a local IVOS prompt tool.

**Objectives:**

To evaluate if prompting for IVOS is currently viewed as a nursing role; to evaluate how confident nurses feel using the local IVOS prompt tool and its suitability for nursing clinical practice; to consider the need for trust-wide nurse engagement and training and to make recommendations to inform antimicrobial stewardship (AMS) nursing practice.

**Methods:**

The local IVOS prompt tool was shown to nurses between November 2022 and March 2023, using a problem-based learning approach to gather nursing views. Nurses were asked to provide anonymous feedback via a short questionnaire, using a five-point Likert scale.

**Results:**

Ninety-eight nurses participated and completed the questionnaire with the following mean scores obtained. Prompting for IVOS was recognized as a nursing role (4.2/5.0); generally, nurses felt confident to use the IVOS tool during the problem-based learning activity (4.1/5.0) and on the ward to assess their patients (4.2/5.0). Qualitative feedback received included ‘*time constraints- another tool [to complete] when there are already many’.* It was deemed that the IVOS tool would be best placed in the clinic room (where IV antibiotics are prepared), as reference aid rather than as an assessment tool requiring formal completion.

**Conclusions:**

Nurses consider IVOS to be within their scope of professional practice and felt confident to use the tool to assess for early switch within an educational and practice setting. Application of the tool in clinical practice is a focus for trust-wide nurse engagement, education and future AMS nursing research.

## References

[1] UKHSA Guidance . National Antimicrobial Intravenous-to-Oral Switch (IVOS) Criteria for Early Switch. 2022. https://www.gov.uk/government/publications/antimicrobial-intravenous-to-oral-switch-criteria-for-early-switch/national-antimicrobial-intravenous-to-oral-switch-ivos-criteria-for-early-switch.

[2] Jenkins A , Anshur D, Postins E et al Poster. How long does it take to administer an intravenous medicine? University Hospitals of Birmingham, 2022.

[3] Carter EJ , Greendyke WG, Furuya EY et al Exploring the nurses’ role in antibiotic stewardship: a multisite qualitative study of nurses and infection preventionists. Am J Infect Control 2018; 46: 492–7. 10.1016/j.ajic.2017.12.01 29395509 PMC6495548

